# Anatomical versus non-anatomical configuration of double coraco-clavicular tunnel technique in acromioclavicular joint reconstruction

**DOI:** 10.1007/s00402-021-03894-0

**Published:** 2021-04-21

**Authors:** Tobias Schöbel, Jan Theopold, Jean-Pierre Fischer, Sabine Löffler, Stefan Schleifenbaum, Pierre Hepp

**Affiliations:** 1 Department of Orthopedic, Trauma and Plastic Surgery, University of Leipzig, Liebigstraße 20, 04103 Leipzig, Germany; 2 ZESBO – Zentrum zur Erforschung der Stuetz- und Bewegungsorgane, Semmelweisstrasse 14, 04103 Leipzig, Germany; 3 Institute of Anatomy, University of Leipzig, Liebigstraße 13, 04103 Leipzig, Germany

**Keywords:** Acromioclavicular joint/injuries/surgery, Arthroscopy/methods, Cadaver, Joint instability/surgery, Ligaments, Articular/surgery, Humans, Joint dislocation, Anatomy

## Abstract

**Purpose:**

Horizontal instability is a common problem after acromioclavicular joint injuries. The aim of this study was to evaluate if there is a difference regarding horizontal stability between an anatomical and a non-anatomical configuration of the double tunnel coraco-clavicular ligament reconstruction of the acromioclavicular joint.

**Methods:**

Thirteen acromioclavicular joints of human cadaveric shoulders in ethanol-glycerin fixation were included in the study and underwent cyclic anterior and posterior translational testing at a load of 70 N using an electromechanical uniaxial testing machine. The shoulders were randomly assigned to the following groups: double coraco-clavicular tunnel technique in an anatomical configuration (DCTa) and double coraco-clavicular tunnel technique in an inverse configuration of the anatomical position (DCTb). The dislocation was recorded with a 3D optical measuring system.

**Results:**

The total horizontal displacement (*p*_10_ = 0.0221; *p*_5000_ = 0.082) was significantly higher for the non-anatomical reconstruction (DCTb) compared to the anatomical reconstruction (DCTa) after every measured amount of cycles. The increase in displacement for DCTb group was overall higher than the increase in displacement for DCTa group but without significance.

**Conclusion:**

Reconstruction of the CC ligaments in an anatomical configuration with two suture devices results in a significantly higher stability of the AC joint in the horizontal plane than reconstruction of the CC ligaments in a non-anatomical configuration. Based on the results of this biomechanical in vitro study, the use of a double coraco-clavicular reconstruction should focus on an anatomically correct position of the suture devices.

## Introduction

Acromioclavicular (AC) joint dislocations are common and add up to 9% of traumatic shoulder injuries [1, 2]. High-grade AC joint dislocations result in a disruption of the AC joint, the coracoclavicular (CC) ligaments and the deltotrapezoid (DT) fascia. The present study focusses on the CC ligaments. In recent years, the surgical treatment for high-grade AC joint dislocation was focused on an anatomical reconstruction of the CC ligaments [3–5]. CC augmentations with synthetic, non-absorbable suture-button-devices were used to mimic the anatomical and biomechanical characteristics of the CC ligaments [6–8]. Unsatisfying results after reconstruction are often based on remaining or recurring instabilities of the horizontal plane of the AC joint [9, 10] Biomechanical and clinical studies have shown the superiority of double button fixations in comparison to single button fixations [6, 11, 12]. However, the exact placement of both constructions might be difficult in clinical practice due to soft tissue coverage, limited surgical exposition and the anatomical variability. To avoid repeated drilling, possibly resulting in a fracture of the coracoid, other trajecteories may be accepted as a compromise [13–15]. Furthermore, no statistically significant difference in the clinical and radiological outcomes between an anatomical reconstruction of the CC ligaments in a V-shaped configuration and CC reconstruction in a parallel configuration was found [16]. To our knowledge, no biomechanical study investigating the influence of the position of the CC ligaments on the stability of the AC joint exists.

Thus, the aim of this in vitro study was to evaluate whether there is a biomechanical difference between an anatomical and a non-anatomical configuration of CC ligament reconstruction using the double tightrope technique. The hypothesis was, that a CC ligament reconstruction using a double coraco-clavicular tunnel technique (DCT) reconstruction in an anatomical configuration would show more stability in the horizontal plane of the AC joint compared to a non-anatomical configuration of the implants. As a non-anatomical configuration, we chose a mirror-inverted reconstruction of the ligaments, thereby attempting to minimize the effects of mutual inhibition of the CC ligaments.

## Material and methods

### Specimen preparation

A total of 13 cadaveric shoulder specimens, 7 right, 6 left shoulders (not matched), from eight human cadavers (3 female and 5 male) were obtained in ethanol-glycerin-fixed condition [17] and were kept at a temperature of 4 °C. The specimens were examined visually before preparation, specimens with visible degeneration or post-injury status of the AC joint were excluded from the examination (*n* = 3). The mean age was 84.8 ± 7.5 years. Soft tissues including the deltotrapezoid fascia were removed from all specimens, leaving only the ligaments and capsule around the AC joint to maintain the original anatomic position of the clavicle. The inferior part of the scapula was secured in a custom block mold to the inferior edge of the glenoid cavity, using alabaster modeling plaster. The presented test model of an AC cerclage was used as a standard [12]. Its effect is not part of the present investigation.

**Fig. 1:**
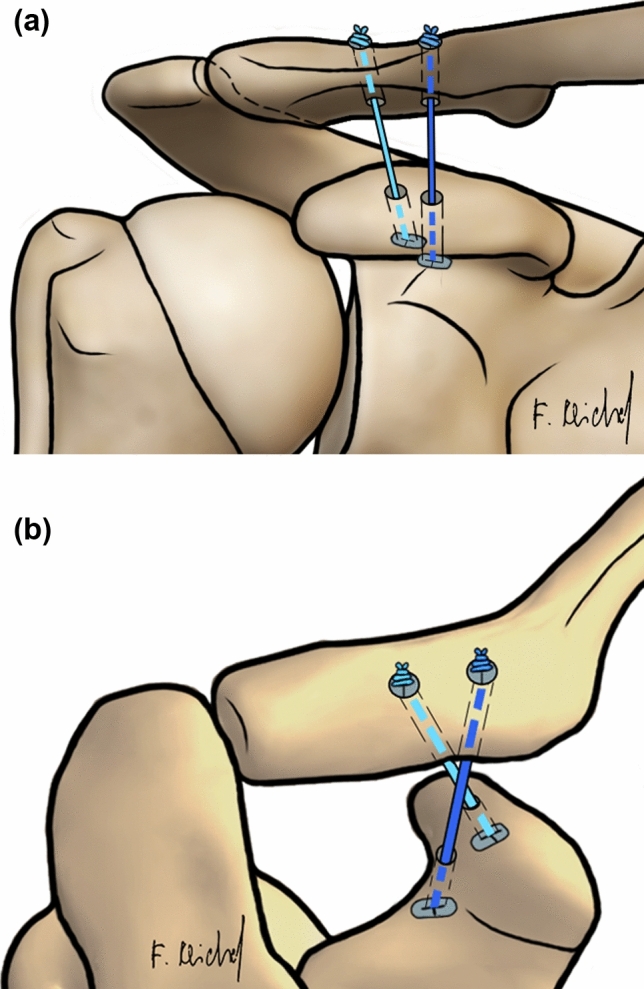
Coracoclavicular ligament reconstruction for the DCT technique with horizontal augmented AC FiberTape® cerclage. **a** Schematic anteroposterior (AP) view on the AC joint. **b** Schematic dorsal view on the AC joint. *DCT* double clavicular tunnel; *AC* acromioclavicular

**Fig. 2:**
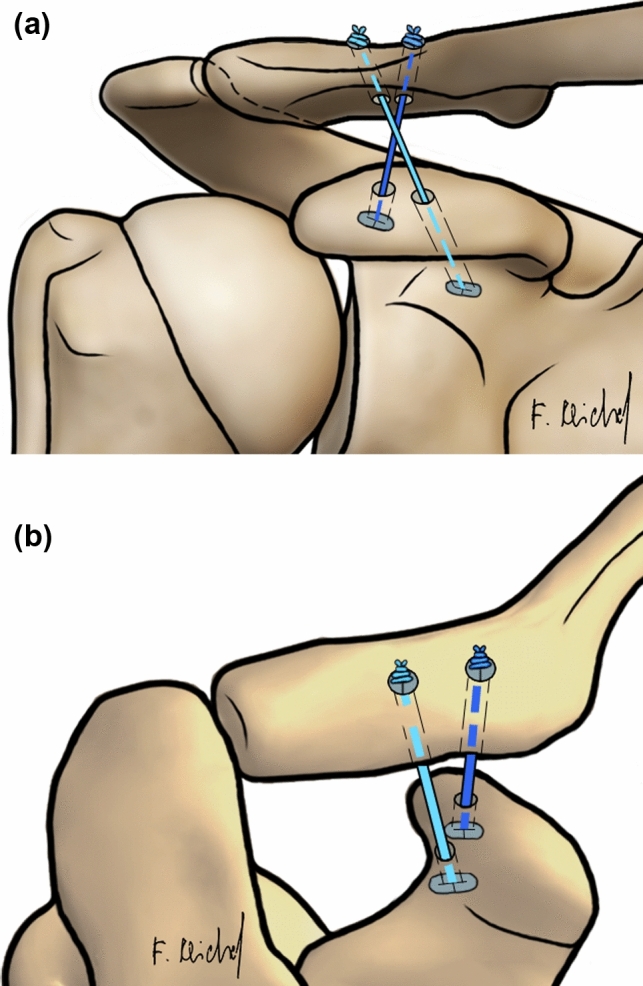
Coracoclavicular ligament reconstruction for the DCT technique in non-anatomical configuration with horizontal augmented AC FiberTape® cerclage. **a** Schematic anteroposterior (AP) view on the AC joint. **b** Schematic dorsal view on the AC joint. *DCT* double clavicular tunnel; *AC* acromioclavicular

Two groups were investigated:

- DCTa group (*n* = 7): DCT technique in an anatomical configuration as a control (Fig. 1).
- DCTb group (*n* = 6) DCT technique in a non-anatomical configuration as the observer group (Fig. 2).

The specimens were randomly assigned to each group.

### Reconstruction techniques

All surgical reconstructions were performed by one experienced surgeon.

The CC ligaments, AC ligaments and the AC capsule were transected. For both techniques, the length of the clavicle had been measured according to Rios et al. [18]. A 20% mark of the total length was set from the lateral edge of the clavicle.

DCTa group: For the DCT technique in an anatomical configuration, a guide was used to drill two 2.4 mm pins from the clavicle to the coracoid process. The first drill (trapezoidal position) started 5 mm lateral of the 20% mark of the clavicle and ended 10 mm dorsal of the ventral edge of the coracoid process. The second drill (conoidal position) started 5 mm medial of the 20% mark of the clavicle, perforating the midpoint of a line between the base of the coracoid process and the neck of the scapula. Both pins were over reamed with the 4 mm cannulated drill. Using passing wires, the suture button devices were passed through the tunnels and tied together over the clavicular button (Fig. 1) [19, 20]. DCTb group: For the DCT technique in a non-anatomical configuration, the configuration was mirrored: The first drill started 5 mm lateral of the 20% mark of the clavicle and ended in the middle of the line between the base of the coracoid process and the neck of the scapula. The second drill started 5 mm medial of the 20% mark of the clavicle and ended 10 mm dorsal of the ventral edge of the coracoid process bottom. Both pins were reamed with the 4 mm cannulated drill. The further procedure was performed as described for DCTa group (Fig. 2).

### Biomechanical testing

**Fig. 3:**
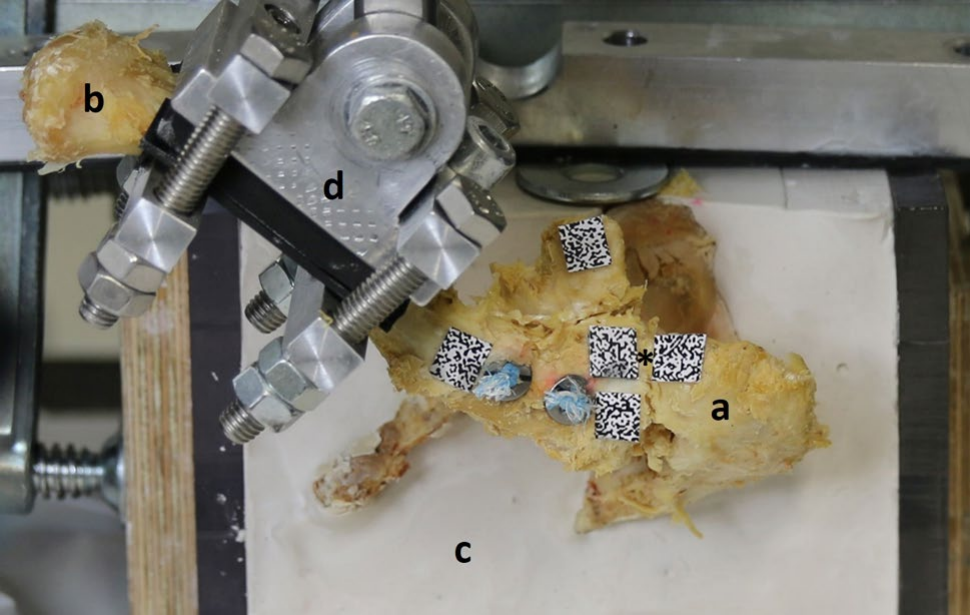
Test setting: Reconstructed specimens (DCT) in the uniaxial testing machine. Acromion (a), Clavicle (b), block mold (c), mounting device (d) and AC joint (*)

The specimens underwent cyclic testing using an electromechanical, uniaxial testing machine (Instron 5566A, Instron GmbH, Darmstadt, Germany). In contrast to other studies [3–5, 8, 21, 22], we used an optical 3D measurement system (Q400 Digital Image Correlation System, LIMESS Messtechnik und Software GmbH, Krefeld, Germany to record images of the resulting displacements (in mm) after a standardized amount of cycles (10, 100, 500, 1000, 2500 and 5000) during the tests for each specimen. This allows a determination of the displacement at the AC-joint without the influence of bending processes of the bone or fixation material. For this purpose, the clavicle length was measured [18] and the midpoint of the clavicle was fixed to the testing machine’s traverse using a customized mounting device. This customized mounting device enabled various angle settings in order to meet the anatomical variation of the cadaveric specimens examined (Fig. 3).

**Fig. 4:**
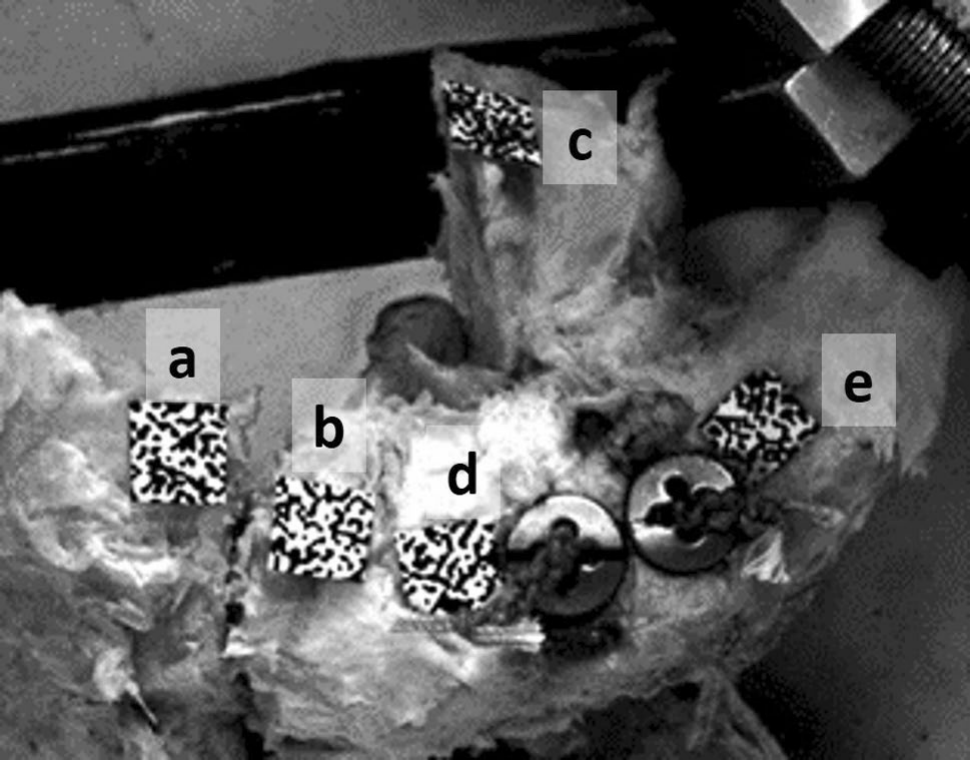
AC joint reconstructed using the DCTa technique with the position of the speckle patterns: 2 mm lateral (a) and 2 mm medial (b) of the AC joint line, coracoid process (c), 10 mm (d) lateral and medial (e) of a 20% mark on the clavicle

The optical 3D measurement system required markers with random speckle patterns that were attached to the surface of clavicle and scapula at relevant points (Fig. 4).

Based on previous studies, a testing load of 70 N was applied cyclically in anterior and posterior direction [4, 8, 21–24]. The first 10 cycles were used for precondition before commencing measurements [4, 8]. In total, 5000 cycles were run to monitor a possible change in the displacement between the groups. The induced traverse motion was force-controlled with a testing speed of 5 mm/s. Throughout the experiment, ethanol-glycerin-solution was applied to prevent tissue dehydration.

**Fig. 5:**
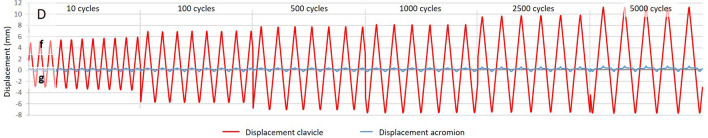
Horizontal displacement in anterior (f) and posterior (g) direction over 5000 cycles

Speckle pattern marks respectively 2 mm lateral and medial of the AC joint line (Fig. 4) were used to measure the AC joint's horizontal displacement. Analysis of optical data from 1932 images per specimen (Fig. 5), obtained throughout the entire course of testing, was performed using the Instra4D software (Dantec Dynamics A/S, Tonsbakken, Denmark).

While alive, all body donors gave their informed and written consent for the donation of their bodies for teaching and research purposes, as part of the body donor program regulated by the Saxonian Death and Funeral Act of 1994. Institutional approval for the use of the post-mortem tissues of the donors was obtained. For this reason, there is no specific number from the ethics committee.

### Statistical analyses

A power analysis was performed, using data from two previous studies with a comparable setup [4, 22]. For an alpha value of 0.05 and a power of 0.90, a minimum of 6 specimens per group were needed. The absolute increase in displacement was calculated by subtracting the displacement measured after 10 cycles of a precondition from the displacement after each measured amount of cycles for each group respectively.

For statistical analysis, SPSS (version 24, SPSS Inc., Chicago, IL, USA) was used. The horizontal displacement of the unpaired specimens and the absolute increase in displacement for the groups was analyzed using the Mann–Whitney-*U*-test. The alpha level was set to *p* < 0.05 for the determination of significance.

## Results

There was neither any hardware nor specimen failure in both groups. Precision of the recorded data was high with a measurement uncertainty of ± 2 µm.

**Fig. 6:**
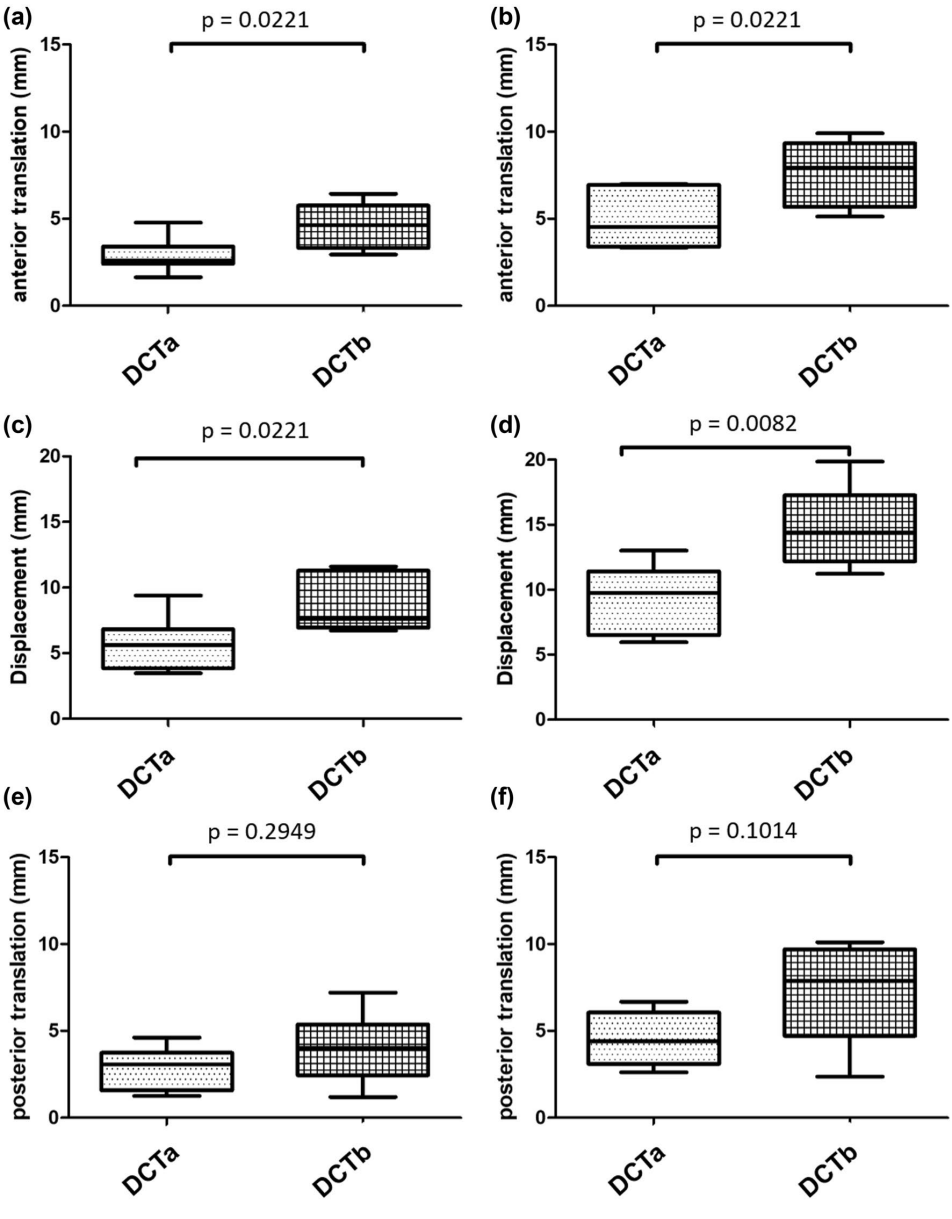
Boxplots for horizontal translation: anterior translation after 10 **(a)** and after 5000 cycles **(b)**, total displacement after 10 **(c)** and after 5000 cycles **(d)**, posterior translation after 10 **(e)** and after 5000 cycles **(f)**

**Fig. 7:**
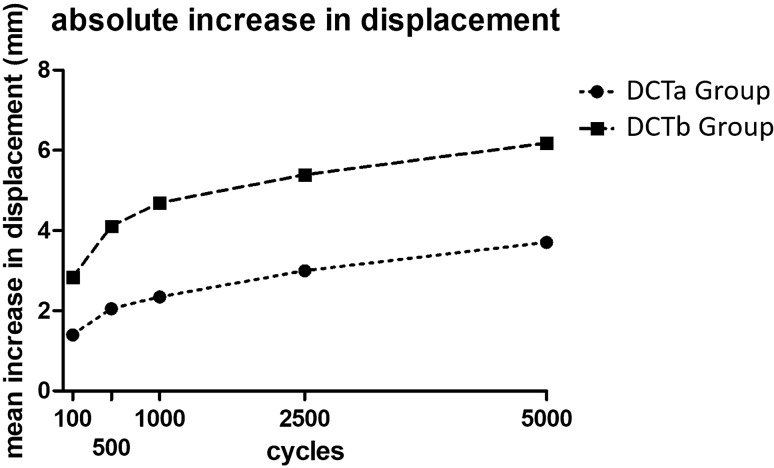
Line graph: Difference of displacement in relation to displacement after precondition between both techniques in dependence of the cycles

The mean anterior translation (*p*_10_ = 0.0221_;_ *p*_100_ = 0.0350; *p*_500_ = 0.0350; *p*_1000_ = 0.0221; *p*_2500_ = 0.0221; *p*_5000_ = 0.0221) and the mean total displacement (*p*_10_ = 0.0221; *p*_100_ = 0.0082; *p*_500_ = 0.0047; *p*_1000_ = 0.0047; *p*_2500_ = 0.0082; *p*_5000_ = 0.0082) for DCTb group was significantly higher after 10, 100, 500, 1000, 2500 and 5000 cycles compared to DCTa group (Table 1, Fig. 6). The specimens showed a total increase of 3.7 mm (relative increase 65%) for the DCTa group and 6.2 mm (relative increase 72%) for the DCTb group after 5000 cycles in comparison to the displacement after 10 cycles. The increase in displacement for DCTb group was overall higher than the increase in displacement for DCTa group but without a statistical significant difference for both groups (*p*_100_ = 0.1375; *p*_500_ = 0.0734; *p*_1000_ = 0.0734; *p*_2500_ = 0.1014; *p*_5000_ = 0.0734) (Fig. 7). For the DCTa group, there was a statistically significant increase in displacement after 50,000 cycles compared to 100 cycles (*p* = 0.0022) but the significance for the DCTb group (*p* = 0.0952).

**Table 1:** Mean horizontal displacement in anterior and posterior direction for the measured numbers of cycles for both groups

Cycles (*n*)	Anatomic reconstruction		Non-anatomic reconstruction	
	Mean displacement (mm)			
	Anterior	Posterior	Anterior	Posterior
10	2.93 ± 1.00*	2.76 ± 1.24	4.61 ± 1.30*	4.02 ± 2.00
100	3.60 ± 1.20*	3.48 ± 1.20	5.62 ± 1.36*	5.71 ± 2.31
500	3.91 ± 1.22*	3.83 ± 1.15	6.12 ± 1.51*	6.58 ± 2.57
1000	4.06 ± 1.24*	3.97 ± 1.14	6.43 ± 1.68*	6.93 ± 2.60
2500	4.53 ± 1.43*	4.15 ± 1.18	6.97 ± 1.65*	7.08 ± 2.82
5000	4.90 ± 1.55*	4.48 ± 1.47	7.65 ± 1.93*	7.21 ± 2.88

*p < 0,05 for both groups

## Discussion

This present biomechanical study shows that the placement of the devices in double tight rope technique in accordance with the anatomic trajectories of the CC ligaments results in significantly higher stability in the horizontal plane of the AC joint compared to a non-anatomical configuration.

The anatomy of the CC ligaments is highly complex and meets the needs of the AC joint. The origin of the lateral trapezoid ligament has a mean distance of 24.9 mm to the lateral edge of the clavicle and it inserts at the top of the coracoid, The mean distance to the origin of the medial conoid ligament is 46.3 mm, it inserts at the bottom of the coracoid [11, 18]. The ligaments are cone-shaped and 2–3 times larger at their clavicular origins than at their insertions at the conoid [25]. Both ligaments have a mean length of 19.4 mm and are oriented in a V-shaped configuration [18, 25–28].

The conoid ligament resists high forces for anterior and posterior loads, the trapezoid ligament counteracts posterior loads [29]. These findings correspond to the suggestion of multiple authors, stating that that the ligaments should not be considered as a single structure in surgical reconstruction [28–30].

The presented study neither evaluates the biomechanical function of the individual ligaments nor the influence of the AC capsule, but the influence of the CC ligaments as a functional unit which provides a higher stability when reconstructed in an anatomical configuration.

Kraus et al. found no statistically significant differences between a CC reconstruction technique placing the coraco-clavicular drill holes in a V-shaped position compared to a CC reconstruction technique placing the drill holes in a parallel position regarding clinical and radiological results two years after surgical treatment [16]. Because of the inherent differences between a clinical and a biomechanical test setting and the differences between the groups tested, these results are only to a limited extend comparable to those from this presented study. Whereas Kraus et al. compared a more anatomical CC reconstruction technique in V-shape to a rather less anatomical, parallel CC reconstruction, we compared an anatomically correct CC reconstruction technique mimicking the position of the conoid and trapezoid ligament to a CC reconstruction using an inverse configuration of the anatomical position. However, our results show that an anatomical position of the CC reconstruction devices may provide more stability in the horizontal plane of the AC joint.

Both groups showed an increased displacement during the cyclic load, without a statistically significant difference between the anatomic and non-anatomic configuration. This elongation is consistent with many biomechanical studies on that matter [12, 31, 32]. Schär et al. compared the horizontal and vertical stability in the AC Joint using Sawbone Specimen [32]. 2 of the 3 groups tested used a double coracoid reconstruction technique, resulting in similar elongation over 1,500 cycles. In the present study, the increase in displacement after 5,000 cycles compared to (after) 100 cycles was approximately equal, confirming their findings.

We assume that due to the specific position and cone shape of the CC ligaments and their V-shaped configuration [18, 25], there is a mutual inhibition of both ligaments, resulting in a higher stability in the horizontal plane of the AC joint. This assumption is supported by a study using a 3-Dimensional Finite Element Model to determine the change in length and tension of the CC ligaments during different positions of shoulder abduction by Seo et al. [33]. They showed that both components of the CC ligament function in a reciprocal mode during shoulder motions. In clinical practice, the effect of coracoid tunnel placement on the stability of the coracoid and AC joint is of importance, especially regarding techniques using the DCT-techniques with at least two coracoclavicular drill holes and exact anatomical placement. This is technically challenging and wrong placement or repeated drilling can result in a failure of the coracoid [13, 34], presumably discouraging surgeons from using these techniques. As an advanced solution, image-free navigated coracoclavicular drilling may enable a precise anatomical positioning of the drill holes [35], reducing the risk of iatrogenic coracoid fractures [36].

This study has the same inherent limitations as other cadaveric studies. (1) The surgical reconstruction technique used remains an approximation to an anatomical reconstruction and does not fully mimic the trajectories of the CC ligaments due to their highly complex anatomy. (2) The anatomical variations of the AC joint and the clavicle affect the fixation of the specimen as well as the surgical positioning and implementation of the suture button devices, thus they may influence the results of the 3D optical measuring system. (3) The biomechanical test setting is not fully transferable into clinical practice. (4) The donors’ age and the ethanol-glycerin fixation: Anatomic coracoclavicular reconstructions are typically performed in young, healthy patients with adequate bone mineral density. However, the specimens were chosen to account for anatomical variations which may not considerably change with age. Furthermore, the anatomy may not be altered by the method of fixation.

## Conclusion

The significantly higher displacement in the horizontal plane and the higher increase of this displacement after a high number of cycles for the non-anatomical CC reconstruction technique in comparison to the anatomical reconstruction implies that an anatomical placement of reconstruction devices for AC joint displacements leads to a more beneficial biomechanical performance, at least in the horizontal plane.

Based on the results of this biomechanical in vitro study, the use of a double coracoid-clavicular reconstruction should focus on an anatomically correct position of the suture devices.
